# Extracellular Vesicles as Players in the Anti-Inflammatory Inter-Cellular Crosstalk Induced by Exercise Training

**DOI:** 10.3390/ijms232214098

**Published:** 2022-11-15

**Authors:** Giulia Catitti, Domenico De Bellis, Simone Vespa, Pasquale Simeone, Barbara Canonico, Paola Lanuti

**Affiliations:** 1 Department of Medicine and Aging Sciences, University “G. d’Annunzio” of Chieti-Pescara, 66100 Chieti, Italy; catittig@gmail.com (G.C.); domenico.debellis@unich.it (D.D.B.); sv85@libero.it (S.V.); p.lanuti@unich.it (P.L.); 2 Center for Advanced Studies and Technology (CAST), University “G. d’Annunzio” of Chieti-Pescara, 66100 Chieti, Italy; 3 Department of Biomolecular Sciences, University of Urbino Carlo Bo, 61029 Urbino, Italy; barbara.canonico@uniurb.it

**Keywords:** extracellular vesicles, inflammation, physical exercise

## Abstract

Extracellular Vesicles (EVs) are circulating particles surrounded by a plasma membrane carrying a cargo consisting of proteins, lipids, RNAs, and DNA fragments, stemming from the cells from which they originated. EV factors (i.e., miRNAs) play relevant roles in intercellular crosstalk, both locally and systemically. As EVs increasingly gained attention as potential carriers for targeted genes, the study of EV effects on the host immune response became more relevant. It has been demonstrated that EVs regulate the host immune response, executing both pro- and anti-inflammatory functions. It is also known that physical exercise triggers anti-inflammatory effects. This review underlines the role of circulating EVs as players in the anti-inflammatory events associated with the regulation of the host’s immune response to physical exercise.

## 1. Introduction

Over the last decade, it has emerged that physical inactivity and sedentary behavior lead to the development of an inflammatory state linked to the onset of several clinical conditions [1,2,3,4]. It is already known that inflammation is aetiologically associated with the pathogenesis of neurological, pulmonary, and cardiovascular diseases (CVD), cancer, and depression [1,5,6,7,8], and a chronic low-grade inflammatory state has been considered a risk predictor factor for some of them [9]. Such an inflammatory state is characterized by high levels of different circulating inflammation markers, such as C-reactive protein (CRP), tumor necrosis factor (TNF), and interleukin-6 (IL-6). Notably, the inactivity induces visceral fat accumulation, triggering the infiltration of the adipose tissue of pro-inflammatory immune cells, further inducing the increased release of adipokines, and the development of a low-grade systemic inflammatory state [7]. The latter, in turn, has been associated with insulin resistance development, tumor growth, neurodegeneration, and atherosclerosis [4,6]. Exercise has anti-inflammatory effects, and, for this reason, regular physical activity may be protective against the development of chronic diseases [1,6,7,8]. 

Three different possible mechanisms have been proposed to explain the anti-inflammatory effects of physical exercise: reduction in visceral fat mass; increased secretion of anti-inflammatory cytokines from contracting skeletal muscles [10,11]; and reduced expression of Toll-like receptors (TLRs) on monocytes and macrophages [12] with the subsequent inhibition of downstream responses, such as the expression of major histocompatibility complex (MHC) and co-stimulatory molecules and the production of pro-inflammatory cytokines [13]. In addition, studies on animal models have underlined that the anti-inflammatory effects induced by physical exercise also rely on other mechanisms, such as the inhibition of monocyte and macrophage infiltration into the adipose tissue and the phenotypic switching of macrophages within the adipose tissue [14]. Although these types of data are difficult to be confirmed in humans, the analysis of human peripheral blood following exercise has shown a decrease in the circulating concentrations of pro-inflammatory monocytes and an increase in the circulating frequencies of regulatory T cells, which have been demonstrated to be involved in the anti-inflammatory effects triggered by physical exercise [15].

In this context, the role of the intercellular crosstalk must be underlined, given that it is a relevant process for the spread of the cell signals to neighboring and/or distant target cells for the regulation of many functions, such as those linked to the immune system, metabolism, survival, repair/regeneration of damaged tissues, and homeostasis maintenance [16]. Intercellular crosstalk is realized by several forms of cell-to-cell communication. Cross-communication among cells may require direct contact with communicating cells (i.e., throughout the gap junctions or tunneling nanotubes). Furthermore, often the intercellular crosstalk is realized by the release from a donor cell of soluble factors (i.e., growth factors, hormones) that act as paracrine or endocrine signals on a recipient cell [16]. More recently, intercellular communication has been associated with the release and the specific loading of extracellular vesicles, small particles secreted in the extracellular milieu theoretically from all cell types [17].

The positive benefits of exercise are believed to be, at least in part, mediated by intercellular crosstalk. The study of EVs in such a context is particularly challenging and, in this review, we analyzed the potential of EVs in the mechanisms by which physical exercise exerts its anti-inflammatory role.

## 2. Extracellular Vesicle-Mediated Intercellular Crosstalk

“Extracellular vesicles” (EVs) is the “umbrella term” including different subtypes of membrane-surrounded particles released in the extracellular milieu by all cell types [18,19]. EV release occurs both in a constitutive and regulated manner; it is induced by Ca^2+^ signaling in response to different stimuli, such as ATP, neurotransmitters, depolarization, thrombin receptor activation, lipopolysaccharides, or cell stress [20,21,22,23,24,25,26]. 

EVs have been involved in intercellular communication, given that upon their release, they are able to pass through biological barriers and therefore can interact with their target cells over long distances [27,28,29]. It is also known that EVs exert their function throughout specific cargoes, consisting of biologically active molecules, such as active enzymes (i.e., the chaperone Hsp70), DNA fragments, mitochondria-derived vesicles, mtDNA, mRNAs, and small RNAs, that they horizontally transfer to target cells [30,31]. Therefore, EVs are specifically loaded to exert their functions on target cells. According to their diameters and the biogenesis mechanisms, EVs have been traditionally classified into apoptotic bodies (larger than 1000 nm), microvesicles released by shedding from the plasma membranes (50–1000 nm), and exosomes (around 100 nm) secreted from multivesicular endosomes [19,32]. Such a classification has been recently revised by the International Society of Extracellular Vesicles (ISEV), establishing the need to use the term EVs for all vesicle subtypes, given that they are overlapping in size and the previously used nomenclature caused confusion [33]. Therefore, the ISEV recommends identifying as small EVs, those smaller than 200 nm, and as large EVs, those with diameters larger than 200 nm [33]. Notably, it has been demonstrated that all body fluids contain mixtures of different EV phenotypes [34,35,36,37,38,39]. It is also known that EVs carry patterns of specific biomolecules related to the phenotypes and the actual status of their parental cells. For these reasons, by studying their phenotypes, it is possible to define their origins [33,37,40,41]. For example, platelet-, endothelial-, and leukocyte-derived EVs can be identified and counted from whole peripheral blood samples [34,37]. Furthermore, circulating EVs, reflecting the physiological and pathophysiological condition of the body, have been identified as reliable biomarkers for liquid biopsy purposes [42,43,44]. Peripheral EV concentrations, as well as their cargo composition, are characterized by a profound inter-subject variability, indicating that their release is a highly dynamic process [45]. 

For these reasons, EVs, with their specific cargoes, are emerging as comprehensive signaling entities mediating adaptive responses over large distances with widespread implications in physiological and pathophysiological events in vivo [39,46,47]. In other words, EVs modulate the functions of target cells by delivering specific cargoes that play a relevant role in intercellular signals [46]. In general, the EV crosstalk has been involved in many different homeostatic processes, such as cell metabolism, maturation, and regeneration events, modulation of the immune system as well as in the activation of blood clotting, and more generally in the processes associated with the dynamic adaptation of cells and tissues to environmental changes [48,49,50,51,52,53,54].

Notably, the EV-mediated immune system modulation is carried out by EVs stemming from immune cells and non-immune cells (mesenchymal stem cells and tumor cells) and may impact both on innate and adaptive immunity [55]. Many reports have demonstrated that lymphocytes, macrophages, dendritic cells (DCs), and natural killer cells (NKs) release EVs with the characteristics of their parental cells [56]. EVs derived from T regulatory (Treg) cells display immunosuppressive effects, given that they carry miRNAs with pro-apoptotic and anti-proliferative functions [57,58]. Therefore, Treg-derived EVs carry miRNA *Let-7d* and suppress interferon-γ (IFN-γ) release and T helper 1 proliferation [59]. Furthermore, EVs stemming from Tregs and expressing CD73 suppress T cell responses and cytokine production [60]. EVs released by CD4+ lymphocytes in general enhance B lymphocyte responses [61], while EVs stemming from CD8+ T cells are related to their parental subtype and its activation status. EVs derived from exhausted CD8+ T cells impair the proliferation, cell activity, and cytokine production of non-exhausted CD8+ lymphocytes, regulating gene expression and metabolic processes [62]. It has been also demonstrated that EVs are more efficient than soluble peptides in transferring antigens between Antigen presenting cells (APCs). Activated macrophages, in fact, release EVs carrying microbial antigens and pathogen-associated patterns that promote macrophage-induced inflammatory responses [63]. Cytotoxic T Lymphocytes (CTLs), NK, and dendritic cells kill their target cells through the release of EVs expressing CD95L [64,65,66]. Macrophages and DCs release EVs carrying enzymes able to synthesize leukotrienes C4 and B4, which mediate pro-inflammatory effects at the inflammation sites [67]. EVs have been implicated in the events regulating the pathogenesis of inflammatory and autoimmune diseases [68,69,70]. The intercellular crosstalk among non-immune and immune cells via EVs has been the focus of more recent scientific research, studying the role exerted by stem cell-derived and tumor-derived EVs on the host immune system. It has been demonstrated that stem cells, and in particular mesenchymal stem cells (MSCs), which roles in regenerative medicine and as immunosuppressive agents are well-established, release EVs. MSC-derived EVs have been demonstrated to produce immunosuppressive effects, throughout the transfer of their cargoes to target cells [71,72,73]. Moreover, mitochondria delivered by EVs participate in immune regulation and exert immunoregulatory effects [74]. On the other hand, it has been shown that tumor-derived EVs may stimulate or suppress the immune system. Given that EVs are enriched in specific tumor antigens, they may stimulate antitumor responses [75,76,77]. However, substantial evidence demonstrates that EVs suppress antigen-specific and non-specific immune responses. Tumor-derived EVs suppress the expression of CD3 ζ-chain, NK cytotoxicity, and CD8+ T cell functions [78]. Tumor-derived EVs also affect APC functions, favoring the generation of myeloid-suppressor cells (MDSCs), which in turn induce the regulatory activity of regulatory T cells, in inhibiting antitumor responses [79,80,81,82]. Furthermore, tumor-derived EVs can directly enhance Treg functions [83]. Moreover, it has been largely demonstrated that many other cell types (platelets, skeletal muscle cells, endothelial cells) release EVs carrying pro-inflammatory or anti-inflammatory cytokines, thus participating in the EV-mediated intercellular exchange of inflammatory or anti-inflammatory factors [34,39,55,84].

## 3. Extracellular Vesicles and Physical Exercise

Physical exercise produces immediate changes in several physiological parameters, such as heart rate, blood pressure, respiration, lactate levels, and circulating cell-free DNA, triggering acute responses. Furthermore, regular exercise initiates beneficial long-term adaptation processes involving muscle metabolism, cardiovascular system responses, and immune modulatory effects [4,85,86,87]. 

In such a context, EVs are merging as signaling entities that can contribute to mediating adaptive responses to physical exercise, possibly because they are implicated in the disposal of cellular waste produced under stress conditions, helping the body to maintain homeostasis, and participating in the immune modulation processes, tissue repair, angiogenesis, and cardio protection [88]. 

It has been demonstrated that the levels of small EVs increased in response to cycling exercise, dropping during the early recovery phase [89,90]. Acute bouts of exercise or short-term training produced an increase in small EV release, even in aged people [91,92,93]. It was also shown that exercise can increase the levels of EVs in diabetic mice after aerobic exercise [94]. 

Furthermore, the EVs released after exercise are enriched in specific markers, such as Programmed cell death 6-interacting protein (Alix), CD81, Annexin A11 (ANAX11), Alpha-actin-4 (ACTN4), and Disintegrin and metalloproteinase domain-containing protein 12 (ADAM12). These EVs have a specific tropism for the liver. Different proteins, and some novel myokines, were modulated in EVs after exercise [95], where myokines are molecules released by skeletal muscle cells and play crucial roles in reducing inflammation and carrying positive effects on lipid and glucose metabolism [96,97,98].

Therefore, exercise produces an increase in peripheral blood EV concentration related to the physiological activation state of the body. However, it is difficult to state the origin of the EVs released by the exercise, even if it may be speculated that, according to published data, the major contributing compartments to the release of EVs after exercise are muscle tissue and cardiomyocytes, endothelial and immune system cells, and platelets [90,95,99,100,101,102,103,104,105,106,107]. 

In cell culture experiments, cardiomyocytes increased EV release under hypoxia [108,109,110]. Therefore, part of the EVs found after exercise could be related to the reduction in the oxygen supply at the tissue level.

Muscle-derived EVs are released into the bloodstream in response to exercise and exert both paracrine and endocrine functions [111,112]. It is known that a total of ∼5% of the circulating small EVs stem from skeletal muscle [110,112], and these data are sustained by the fact that skeletal muscle cells release higher numbers of EVs than adipocytes [112]. Furthermore, using reporter mice, it was demonstrated that skeletal muscle EVs are released directly into circulation [112]. Those EVs seemed to be released upon aerobic exercise rather than by resistance exercise [113]. The release of muscle-derived EVs into circulation after exercise has been largely demonstrated by the increase in muscle- specific miRNAs in EVs [110,114]. Muscle-derived EVs participate in the systemic antioxidant defense [115]. Skeletal muscle-derived EVs exert their functions at long distances, passing across the biological barriers, given that, after their intraperitoneal injection in mice, they have been found within many different cell compartments, such as skeletal muscle, brain, liver, heart, lungs, gastrointestinal tract, spleen, kidney, and pancreas cells [116]. Additionally, it appears that skeletal muscle EVs contribute to the crossover effects of unilateral exercise [117]. Twenty-four hours after the injection of green fluorescent protein-labeled skeletal muscle EVs into the right tibialis anterior of mice, fluorescence was detected in the right quadriceps and the left tibialis anterior [116]. It was largely demonstrated, both in humans and in animal models, that aerobic physical exercise exerts beneficial effects on adipose tissue and, in general, on whole-body metabolism. In detail, both acute and chronic exercise increase catecholamine sensitivity. A single bout of resistance exercise increases adipocyte lipolysis and muscle fatty acid oxidation and such lipolytic response was impaired in obese men [118]. In response to mechanical overload, skeletal muscle cells release EVs carrying *miR-1*. Those EVs act on epidydimal white adipose tissue, where *miR-1* promotes adrenergic signaling and lipolysis [114], and, based on these results, a clinical trial has been set up (NCT04500769).

However, capillarization is a fundamental process that influences exercise resistance, performance, muscle mass preservation, and muscle insulin response. Many factors may negatively or positively impact on skeletal capillarization. Among them, vascular endothelial growth factor (VEGF), produced by different cell subtypes, stimulates the formation of blood vessels, playing a crucial role in skeletal muscle capillarization. Of note, VEGF was not detected in EVs stemming from skeletal muscle cells, moreover, EVs cannot induce any VEGFR2 phosphorylation in recipient endothelial cells. Conversely, skeletal muscle-derived EVs induce the increase in the pro-inflammatory cytokine *interleukin-8* (*IL8)* and of *Angptl4* (*Angiopoietin-like 4*) without affecting *Ang2/Ang1* (*angiopoietin 2/angiopoietin 1*), *Mcp1* (*monocyte chemoattractant protein 1*), *VEGF*, and *BDNF* (*brain-derived neurotrophic factor*) mRNA expression [119]. These data suggest that skeletal muscle-derived EVs activate an angiogenetic modulating mechanism independent from VEGF-signaling. This mechanism may be instead attributed to the production of reactive oxygen species (ROS) and to the activation of nuclear factor-κB (NF-κB) signaling in endothelial cells. The characterization of miRNAs carried by skeletal muscle-derived EVs has revealed that they are muscle-specific (*miR-133a* and *miR-206*) and related to pro-angiogenic processes (*miR-15*, *miR-16*, *miR-126*, *miR-130a*, *miR-210*, *miR-221*, *miR-222*, *miR-378*, *miR-503*, and *let7f*). Those miRNAs can be transferred to endothelial cells, inducing the overexpression of *miR130a* with the consequent downregulation of anti-angiogenic targets, such as *mesenchyme homeobox 2* (*Gax*). Furthermore, an increased EV production was demonstrated in oxidative more than in glycolytic muscles and this evidence was consistent with a greater capillarization of oxidative muscles [119]. As stated, together with EVs of muscle origins, endothelial-derived EVs are also released under moderate endurance exercise [99]. It is known that exercise promotes angiogenesis through the upregulation of liver-derived EVs carrying *miR-122-5p*, which enhances fatty acid utilization by targeting 1-acyl-sn-glycerol-3-phosphate acyltransferase alpha (AGPAT1) in endothelial cells, highlighting its therapeutic potential in tissue repair [120].

Furthermore, platelets contribute to the release of EVs after training, given that it was demonstrated that platelet-derived EVs, associated with pro-coagulant and regenerating functions, increased when subjects underwent strenuous exercises [99,101,104,105,106,107].

## 4. Anti-Inflammatory Roles of Exercise-Related EVs

**Figure 1:**
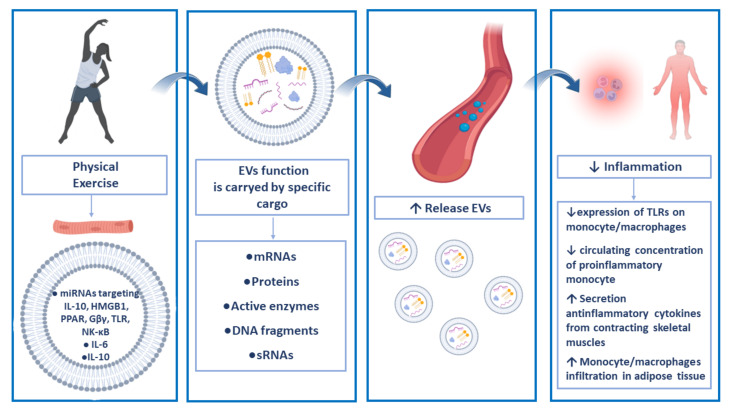
Physical exercise and anti-inflammatory effects of EVs. Physical exercise induces the release of EVs of different origins. It has been demonstrated that exercise training induces the secretion of EVs stemming from skeletal muscle cells that carry anti-inflammatory signaling molecules (miRNAs and cytokines). Once released, (leukocyte-, muscle-, and platelet-derived EVs) EVs act locally and systemically reaching target tissues throughout blood circulation. EVs produced by physical exercise therefore act as anti-inflammatory agents.

The anti-inflammatory effects elicited by EVs have been largely demonstrated [121,122,123,124]. The EV contribution to exercise-induced improvements in systemic inflammation was also analyzed, and muscle and peripheral blood cells have been implicated in such a mechanism (Figure 1). Muscle-derived EVs improve inflammatory signaling and increase endothelial cell proliferation, migration, and tube formation through the activation of the NF-κB pathway [119]. Sullivan and collaborators demonstrated that obesity alters skeletal muscle-derived EV miRNAs, affecting mRNA targets belonging to the growth pathways (cardiac hypertrophy, *Wnt/β-catenin*, *Phosphoinositide 3-kinases/RAC-alpha serine/threonine-protein kinase, PI3K/AKT*, *Insulin-like growth factor 1, IGF-1*, and *Phosphatase and tensin homolog, PTEN*) and inflammation signaling (*Pigment endothelium-derived factor, PEDF*, *death receptor*, and *Gα_i_*). In addition, one week of concurrent aerobic and resistance exercise training altered skeletal muscle-derived small EV miRNAs targeting mRNA related to inflammation (*Interleukin-10*, *IL-6*, role of macrophages, *Toll-like receptor*, *HMGB1*, and *NF-κB*), growth (cardiac hypertrophy and *Gβγ*), and metabolism (*Peroxisome proliferator-activated receptor, PPAR*), indicating an overall reduction in inflammation. In particular, the activation of PPAR signaling increases lipid metabolism, exercise tolerance, and mitochondrial biogenesis via peroxisome proliferator-activated receptor gamma coactivator 1-alpha (PGC1-α). Skeletal muscle *Wnt3a*, *Wnt5a*, and *Wnt7a*, and *IGF-1* mRNAs are all reduced in obese subjects; moreover, one week of concurrent exercise training reduces, in skeletal muscle cells, mRNA expression levels of *Jun*, *Fos*, and *IL-8* by approximately 25%, 65%, and 50%, respectively, in both lean and obese individuals [125]. Exercise training also induces the release and the loading of EVs acting as anti-inflammatory agents on skeletal muscles. Contracting muscles may directly release IL-6. It has been demonstrated that exercise-induced muscle-derived IL-6 exerts anti-inflammatory effects, inhibiting TNF-α, IL-10, and IL-1β activities, and protecting against TNF-induced insulin resistance [96,126,127]. The regulation of IL-6 by physical exercise has been widely investigated in the last years and IL-6 was found to be the main cytokine involved in exercise physiology. IL-6 levels rise to 100-fold (especially during intense and prolonged exercise) after physical activity, but they drop rapidly during the following rest period [128], and IL-6 has been defined as a myokine (a cytokine secreted from active skeletal muscle) [96]. The IL-6 produced by physical exercise therefore appears to have a purely anti-inflammatory role. This role seems to be mediated through the induction of IL-1Ra and IL-10 as well [128]. EVs released after exercise also carry meteorin-like protein [129], a molecule able to stimulate the release of many anti-inflammatory cytokines [130].

In animal models undergoing aerobic, acrobatic, resistance, or the combination of previous exercise typologies, the cargo analysis of the EVs released after the training was carried out, demonstrating that aerobic exercise produced an increase in brain-derived neurotrophic factor (BDNF) and Interleukin- 1β (IL-1β) in aged rats, while acrobatic and combined exercises decreased the IL-1β content within EVs from adult rats. For these reasons, it has been hypothesized that the aforementioned changes may be associated with the previously observed reduction in mortality rate and improvement in memory performance [131].

It is also known that altogether EVs released after exercise, carrying IL-10 and the other anti-inflammatory cytokines, produce systemic effects, and exert their beneficial actions on the brain, directly regulating central inflammation, therefore protecting against inflammation-dependent neurological diseases [132,133].

## 5. Conclusions

This review underlines the role of circulating EVs as players in the anti-inflammatory events associated with the regulation of the host immune response to physical exercise. Many large epidemiological studies have demonstrated that physical inactivity is related to the occurrence of cardiovascular diseases (CVD), the leading cause of death in modern societies. For these reasons, studying the mechanisms related to such a phenomenon may suggest new strategies to improve the effects of exercise on inflammation processes implicated in the development of CVD and other chronic pathologies. In this context, EVs may play a central role and, even if more studies are needed to better identify the specific EV cargo modifications triggered by physical exercise, EVs are promising systemic carriers of anti-inflammatory messages.

## Data Availability

Not applicable.
